# CD4 Counts at Entry to HIV Care in Mexico for Patients under the “Universal Antiretroviral Treatment Program for the Uninsured Population,” 2007–2014

**DOI:** 10.1371/journal.pone.0152444

**Published:** 2016-03-30

**Authors:** Alfonso C. Hernández-Romieu, Carlos del Rio, Juan Eugenio Hernández-Ávila, Hugo Lopez-Gatell, José Antonio Izazola-Licea, Patricia Uribe Zúñiga, Mauricio Hernández-Ávila

**Affiliations:** 1 Department of Global Health, Rollins School of Public Health, Emory University, Atlanta, GA, United States of America; 2 Department of Medicine, Emory University School of Medicine, Atlanta, GA, United States of America; 3 Center for AIDS Research, Emory University, Atlanta, GA, United States of America; 4 National Institute of Public Health (INSP), Cuernavaca, Mexico; 5 National Center for Prevention and Control of HIV/AIDS (CENSIDA), Mexico City, Mexico; 6 Joint United Nations Programme on HIV/AIDS (UNAIDS), Evaluation and Economics Division, Geneva, Switzerland; FIOCRUZ, BRAZIL

## Abstract

In Mexico, public health services have provided universal access to antiretroviral therapy (ART) since 2004. For individuals receiving HIV care in public healthcare facilities, the data are limited regarding CD4 T-lymphocyte counts (CD4e) at the time of entry into care. Relevant population-based estimates of CD4e are needed to inform strategies to maximize the impact of Mexico’s national ART program, and may be applicable to other countries implementing universal HIV treatment programs. For this study, we retrospectively analyzed the CD4e of persons living with HIV and receiving care at state public health facilities from 2007 to 2014, comparing CD4e by demographic characteristics and the marginalization index of the state where treatment was provided, and assessing trends in CD4e over time. Our sample included 66,947 individuals who entered into HIV care between 2007 and 2014, of whom 79% were male. During the study period, the male-to-female ratio increased from 3.0 to 4.3, reflecting the country's HIV epidemic; the median age at entry decreased from 34 years to 32 years. Overall, 48.6% of individuals entered care with a CD4≤200 cells/μl, ranging from 42.2% in states with a very low marginalization index to 52.8% in states with a high marginalization index, and from 38.9% among individuals aged 18–29 to 56.5% among those older than 50. The adjusted geometric mean (95% confidence interval) CD4e increased among males from 135 (131,142) cells/μl in 2007 to 148 (143,155) cells/μl in 2014 (p-value<0.0001); no change was observed among women, with a geometric mean of 178 (171,186) and 171 (165,183) in 2007 and 2014, respectively. There have been important gains in access to HIV care and treatment; however, late entry into care remains an important barrier in achieving optimal outcomes of ART in Mexico. The geographic, socioeconomic, and demographic differences observed reflect important inequities in timely access to HIV prevention, care, and treatment services, and highlight the need to develop contextual and culturally appropriate prevention and HIV testing strategies and linkage programs.

## Introduction

Mexico is an upper-middle income country with a concentrated HIV epidemic.[1] In 2012, the estimated number of people living with HIV (PLHIV) aged 15–49 was 147,137, with a mean estimated prevalence of 0.15% (0.07% among women and 0.24% among men).[2] Men who have sex with men (MSM) and transgender women have the highest HIV prevalence, estimated at 17% and 19.8%, respectively.[3, 4]

Timely diagnosis of HIV, prompt linkage and retention in care, and initiation of antiretroviral therapy (ART) are crucial in achieving optimal health outcomes among persons living with HIV. PLHIV who enter care and initiate ART with lower CD4+ lymphocyte counts (CD4) have increased morbidity and mortality, are less likely to achieve viral suppression, and have slower CD4 recovery.[5–10] Late diagnosis, delayed entry into care, and late initiation of ART are also associated with increased transmission of HIV.[11, 12] Furthermore, entering care with high CD4 counts results in fewer quality-adjusted life-years lost for PLHIV and lower HIV related treatment expenditures.[13, 14]

Evidence from international reports and studies shows that a large portion of PLHIV enter care and initiate ART well below the 500 CD4 cells/μl threshold established by 2013 WHO guidelines.[15] A recent meta-analysis of data from Sub-Saharan Africa estimated a mean CD4 at presentation to care (CD4e) of 309 cells/μl in 2012 [16]; an analysis of Asian cohorts yielded a median CD4e of 302 cells/μl in 2011[17]; and in cohorts of the United States and Canada the median CD4e was 317 cells/μl in 2007.[18]

For Mexico, information regarding late entry into HIV care is limited and shows considerable gaps. A study from one tertiary care hospital in Mexico City, based in a sample of 429 patients studied between 2001–2008, recorded a median CD4 of 149 cells/μl, and showed that 75% initiated ART with a CD4 < 200 cells/μl or with an AIDS-defining illness.[19] Another report from Tijuana (a border city with the US) showed that among a sample of 362 AIDS patients identified in two HIV/AIDS public clinics, 43.2% were late testers.[20] Timely data from CD4 surveillance are needed to monitor both late diagnosis and the performance of the Mexican national HIV program to guide strategies to optimize outcomes of universal ART access.

Since 1997, Mexico has provided access to HIV care and treatment in social security institutions covering employees in the formal labor market. Funding to cover HIV care for the unemployed or populations in the informal sector became available in 2003, through a voluntary government-subsidized health insurance program providing limited healthcare and financial protection; this program is called Seguro Popular (SP).[21, 22] After SP implementation, the number of previously uninsured PLHIV receiving ART increased from 14,447 in 2006 to 93,166 in 2014.[23] HIV care covered by SP is monitored through the ART administration, logistics, and surveillance system (SALVAR for its acronym in Spanish). SALVAR, established in 2006, only includes data for PLHIV enrolled in and receiving care through SP at public health facilities, and covers 62% of PLHIV receiving HIV care in Mexico at the end of 2014. Since 2009, HIV treatment guidelines in Mexico have recommended starting ART at a CD4 ≤350 cells/μl; these were modified in 2014 to <500 cells/μl.[24, 25]

We retrospectively analyzed SALVAR data of PLHIV who received HIV care at public health facilities from 2007 to 2014 to estimate time trends in CD4e and to compare demographic and geographic characteristics among SP beneficiaries in Mexico with various levels of CD4e.

## Methods

### Data source

Used by all 173 prevention and treatment centers located in the 31 states and the Federal District, SALVAR is a web-based secure information system that connects HIV/AIDS public clinics funded by SP with the National Center for HIV Prevention and Control (CENSIDA). Information includes nominal records on all PLHIV receiving care at public clinics and hospitals regardless of whether they are receiving ART. Health personnel at participating institutions report demographic, biometric, clinical, and ART prescription data for individuals in care. A patients' consent to register his or her personal data is obtained by a physician at the first visit, as mandated by law.[26] For this analysis, CENSIDA provided the authors with an anonymized de-duplicated set of demographic data as well as biometric and ART prescription data that included the dates of biometric measurements and initiation of ART therapy. Records of uninsured individuals receiving care prior to the implementation of SALVAR were batch-transferred into the system during 2006, its first year of operation. Because SP began covering costs for CD4 and HIV-1 RNA (i.e., viral load) testing in 2009, tests prior to that year were performed and reported less regularly. The Emory University Institutional Review Board approved the study (protocol #00058269). All data are stored on restricted and secure databases, with adherence to the Mexican Federal Law on the Protection of Personal Data.

### Study population

We identified first entry into HIV care as the date of the first recorded CD4. SALVAR included data on 93,501 adults aged ≥18 years of age who entered care from 2004–2014, of whom 93,297 (99.8%) had at least one CD4 count recorded. Among individuals with a non-missing CD4 count, 76,820 (81.1%) had a CD4 recorded between 2007 and 2014. To minimize the inclusion of non-ART naïve individuals, we excluded patients with a date of entry into care prior to 2007. Among individuals with a first CD4 on or after 2007, the following inclusion criteria were used: 1) the first recorded CD4 count of individuals who were registered as not receiving ART in SALVAR, and 2) individuals registered as receiving ART if the first recorded CD4 measurement was prior to, or up to one month after, the recorded date of ART initiation.

### Measures and variables of interest

The primary outcome of interest was the annual cross-sectional trend in the geometric mean CD4e at entry into treatment. Potential predictors included sex, age, and state where care was provided. To incorporate socioeconomic differences among states where patients received care, states were categorized according to the 2010 state Marginalization Index (MI). MI was developed by the Mexican National Population Council to compare human, social, and economic development over time and across the 31 states and the Federal District (i.e., Mexico City).[27] The MI uses population estimates of access to education, housing conditions, income, and rurality (i.e., communities with less than 5,000 inhabitants) to calculate an overall index score which is grouped into five categories, ranging from very low to very high marginalization.

### Statistical analysis

The population entering care each year by sex, age group, state MI, and by CD4e is described. CD4e were described using geometric means, calculated by back-transforming the natural logarithm of CD4e, and categorically using clinically relevant cut-points for prevention of opportunistic infections and initiation of ART.[15, 28] Geometric means rather than arithmetic means were used because of the highly skewed distribution of CD4e. We used the Cochran-Armitage trend test to assess changes in the distribution of sex over time, and used Cuzick’s trend test for ordered variables to assess changes in distributions of age and state MI. Bivariate logistic regression with odds ratios (OR) and their 95% confidence intervals (CI) was used to assess changes in the Male to Female (M:F) ratio by year of entry into care, and to compare the proportion of males to females by state MI.

We assessed sex-specific trends of the natural logarithm of CD4e from 2007–2014 by using a linear regression model with individual observations. We added a single knot linear spline for the year 2011 and its interaction with sex as covariates to allow statistical testing for changes in the slope of temporal trends. We chose 2011 because exploratory data analyses showed a decreasing trend in CD4e after this year. We estimated the standard errors of parameters and predicted values using a bootstrap method with 200 repetitions to accommodate for possible model misspecifications due to skewed CD4e data.[29]

To explore variation by sex, age, and marginality index we fitted a second linear model that included the natural logarithm of CD4e counts as dependent and dummy variables for sex, age and state MI, as well as their 4-way interaction terms with temporal splines as predictors. The result of this model parametrization is a set of sub-models with an intercept and slope for each age-group-gender-MI subpopulation and period, allowing us to individually test the significance of the respective CD4e trends on both sides of the knot of the linear spline in 2011 (t = 1 and t = 2). Thus, the linear combination of the respective coefficients of the model represents the annual percentage of change in the geometric mean of the initial CD4e counts, shown in the following equation: $\%\;annual\;change\;in\;initial\;CD4e\;counts={(e}^{\beta_{i}}-1)*100$, where β_i_ is a linear combination of the coefficients. The results of the models are presented in graphic and tabular formats showing the percent change and 95% confidence intervals (CI) in CD4e before and after 2011. We performed a likelihood ratio (LR) test to assess the significance of the interaction terms and constructed a table of likelihood ratios with the main effects as the smallest model and adding all of the first, second, third and fourth way interaction terms. We decided that, for simplicity and direct interpretation, results should reflect the annual percent change in each of the groups. In addition, to increase the ability of others to interpret and use our results, we grouped independent variables according to the HIV/AIDS control program’s relevant population groups.

We performed another likelihood ratio to test whether a multilevel model, with observations clustered on state MI, would better fit the data; the LR test was not statistically significant and therefore fit linear models for parsimony.

Statistical hypotheses were tested using an α = 0.05 based on 2-tailed tests. We calculated 95% CI for all geometric mean CD4e. All analyses were performed using SAS version 9.4 (SAS Institute; Cary, NC) and STATA version 13 (Stata Corp; College Station, TX).

## Results

A total of 66,947 (87.1%) PLHIV who entered care from 2007–2014 met the inclusion criteria. The annual number of new individuals entering HIV care increased from 6,211 in 2007 to 10,860 in 2014 (Table 1). Men accounted for 79% of all adults entering care, and the proportion of men increased monotonically during the period studied (OR 1.05, p-value <0.0001; not shown), with the M:F increasing from 3.0 in 2007 to 4.3 in 2014. The median age (inter-quartile- range) was 34 (28–41) in 2007 and 32 (26–40) in 2011. Thirty-seven percent of PLHIV entering care from 2007 to 2014 received care in high or very high marginalization states. The distribution of patients by state MI did not change significantly during the period studied (p-value 0.07). The M:F was inversely related to the marginalization index (OR 0.78, CI [0.77, 0.79]; p-value<0.0001), from 8.2 in very low MI states to 2.4 in very high MI states (data not shown).

**Table 1:** Demographic characteristics of individuals entering health care program, SALVAR, Seguro Popular, Mexico 2007–2014.

	Total	2007	2008	2009	2010	2011	2012	2013	2014	
	n (%)	n (%)								*P*‡
Total	66947 (-)	6211 (9.3)	6944 (10.4)	6949 (10.4)	7329 (11)	8244 (12.3)	10047 (15)	10,363 (15)	10860 (16.2)	
Sex*										<0.0001
Female	13971 (20.9)	1538 (24.8)	1647 (23.7)	1511 (21.7)	1571 (21.4)	1750 (21.2)	2036 (20.3)	1862 (18.0)	2056 (18.9)	
Male	52976 (79.1)	4673 (75.2)	5297 (76.3)	5438 (78.3)	5758 (78.6)	6494 (78.8)	8011 (79.7)	8501 (82.0)	8804 (81.1)	
M:F ratio	3.8	3.0	3.2	3.6	3.7	3.7	3.9	3.9	4.3	
Age (years)										<0.0001
median (IQR)	33 (26–41)	34 (28–41)	34 (27–41)	33 (27–40)	33 (27–41)	33 (27–41)	32 (26–40)	32 (26–41)	32 (26–40)	
18–29	25205 (37.7)	1995 (32.1)	2395 (34.5)	2536 (36.5)	2623 (35.8)	3018 (36.6)	4003 (39.8)	4145 (40.0)	4490 (41.3)	
30–49	35810 (53.5)	3607 (58.1)	3957 (57.0)	3825 (57.0)	4067 (55.5)	4472 (54.3)	5192 (51.7)	5304 (51.2)	5386 (49.6)	
≥50	5932 (8.9)	609 (9.8)	592 (8.5)	588 (8.5)	639 (8.7)	754 (9.2)	852 (8.5)	914 (8.8)	984 (9.1)	
State MI†										0.07
Very low	13470 (20.1)	1131 (18.2)	1171 (16.9)	1364 (19.6)	1324 (18.1)	1544 (18.7)	2239 (22.3)	2441 (23.6)	2256 (20.8)	
Low	20182 (30.2)	1898 (30.6)	2332 (33.6)	2165 (31.2)	2439 (33.3)	2424 (29.4)	2893 (28.8)	2992 (28.9)	3039 (28.0)	
Moderate	8533 (12.8)	844 (13.6)	937 (13.5)	869 (12.5)	983 (13.4)	1098 (13.3)	1201 (12.0)	1197 (11.6)	1404 (12.9)	
High	17171 (25.7)	1866 (30)	1756 (25.3)	1760 (25.3)	1760 (24)	2178 (26.4)	2539 (25.3)	2500 (24.1)	2812 (25.9)	
Very high	7591 (11.3)	472 (7.6)	748 (10.8)	791 (11.4)	823 (11.2)	1000 (12.1)	1175 (11.7)	1233 (11.9)	1349 (12.4)	

SALVAR: antiretroviral drug administration, logistics and surveillance system; IQR: inter-quartile range; MI: marginalization index

*Includes 37 reported as transgender individuals, 36 of whom were biologically male and 1 female

^†^MI of the state of residence was classified according to the CONAPO marginalization index 2010, available at: http://www.conapo.gob.mx/en/CONAPO/Indices_de_Marginacion_2010_por_entidad_federativa_y_municipio

^‡^P-value for linear trend by sex category using Cochran Armitage test, other tests for linear trends using Cuzick's trend test for ordered groups

Between 2007 and 2014, 48.6% of PLHIV entered care with CD4e <200 cells/μl, with 18% entering care with CD4e <50 cells/μl; the geometric mean (95% CI) was 158 (157,160) (Table 2). Between 53–54% of PLHIV in high and very high MI states entered care with a CD4e <200 cells/μl, as did 56.5% of PLHIV 50 or more years of age.

**Table 2:** CD4 T-lymphocytes values at first entry into health care program, SALVAR, Seguro Popular, Mexico, 2007–2014.

	CD4 (cells/μl) at entry into care						
	<50	50–99	100–199	200–349	350–499	≥500	geometric mean (95%CI)
		n (%)					
Total	12183 (18.2)	8299 (12.4)	12079 (18.0)	14220 (21.2)	9443 (14.1)	10723 (16.0)	158 (157–160)
Year of entry into care							
2007	1150 (18.5)	808 (13.0)	1295 (20.9)	1357 (21.9)	773 (12.5)	828 (13.3)	148 (143,152)
2008	1353 (19.5)	906 (13.1)	1338 (19.3)	1544 (22.2)	897 (12.9)	906 (13.1)	144 (140,149)
2009	1199 (17.3)	912 (13.1)	1294 (18.6)	1471 (21.2)	949 (13.7)	1124 (16.2)	161 (157,166)
2010	1286 (17.6)	966 (13.2)	1284 (17.5)	1480 (20.2)	1077 (14.7)	1236 (16.9)	161 (157,166)
2011	1304 (15.8)	994 (12.1)	1452 (17.6)	1735 (21.1)	1190 (14.4)	1569 (19.0)	176 (172,181)
2012	1764 (17.6)	1209 (12.0)	1710 (17.0)	2094 (20.8)	1480 (14.7)	1790 (17.8)	167 (163,171)
2013	2103 (20.3)	1251 (12.1)	1808 (17.5)	2170 (20.9)	1461 (14.1)	1570 (15.2)	149 (145,153)
2014	2024 (18.6)	1253 (11.5)	1898 (17.5)	2369 (21.8)	1616 (14.9)	1700 (15.7)	158 (154,161)
Sex							
Female	2113 (15.1)	1537 (11.0)	2514 (18.0)	3082 (22.1)	2093 (15.0)	2632 (18.8)	182 (178,185)
Male	10070 (19.0)	6762 (12.8)	9565 (18.1)	11138 (21.0)	7350 (13.9)	8091 (15.3)	152 (151,154)
Age (years)							
18–29	3432 (13.6)	2385 (9.5)	3971 (15.8)	5965 (23.7)	4443 (17.6)	5009 (19.9)	197 (195,200)
30–49	7502 (21.0)	5005 (14.0)	6917 (19.3)	7091 (19.8)	4359 (12.2)	4936 (13.8)	141 (139,144)
≥50	1249 (21.1)	909 (15.3)	1191 (20.1)	1164 (19.6)	641 (10.8)	778 (13.1)	136 (132,140)
State MI*							
Very low	1940 (14.4)	1477 (11.0)	2260 (16.8)	3170 (23.5)	2223 (16.5)	2400 (17.8)	185 (181,189)
Low	3726 (18.5)	2484 (12.3)	3491 (17.3)	4202 (20.8)	2835 (14.1)	3444 (17.1)	160 (157,163)
Moderate	1470 (17.2)	975 (11.4)	1461 (17.1)	1737 (20.4)	1234 (14.5)	1656 (19.4)	172 (167,176)
High	3739 (21.8)	2290 (13.3)	3235 (18.8)	3466 (20.2)	2166 (12.6)	2275 (13.3)	136 (133,138)
Very high	1308 (17.2)	1073 (14.1)	1632 (21.5)	1645 (21.7)	985 (13.0)	948 (12.5)	151 (147,155)

SALVAR: antiretroviral drug administration, logistics and surveillance system; MI: marginalization index

*MI of the state of residence was classified according to the CONAPO marginalization index 2010, available at: http://www.conapo.gob.mx/en/CONAPO/Indices_de_Marginacion_2010_por_entidad_federativa_y_municipio

**Fig 1:**
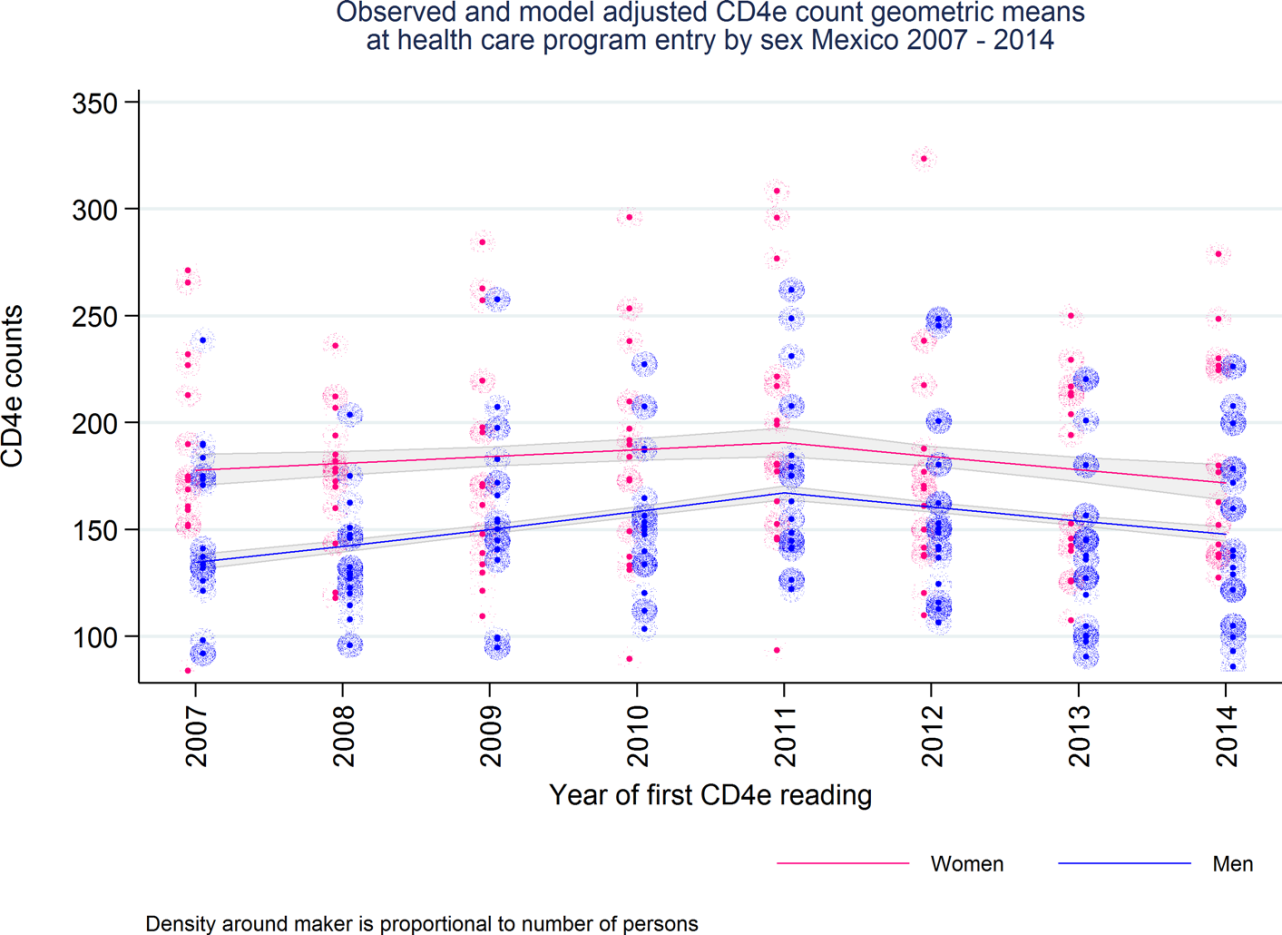
CD4e counts at health care program entry decline after 2011.

Fig 1 summarizes the estimations derived from a non-interaction sex-specific adjusted log linear model to predict the annual geometric mean of the CD4e cell count. During the study period, the annual CD4e cell count displayed a U-inverted shape, with a turning point in 2011. The magnitude of the increase and decrease varied by sex (Fig 1). For women, the annual geometric mean CD4e cell count increased by 1.8% (95% CI: 0.2%, 3.4%), from 178 cells/μl in 2007 to 191 cells/μl in 2011; afterward, CD4e declined annually by 3.4% (95% CI: 1.4%, 5.4%) reaching an annual geometric mean CD4e of 171 cells/μl in 2014. A larger increase in geometric mean CD4e cell counts was observed among men, with an annual increase of 5.6% (95% CI: 4.6%, 6.5%). The CD4e was135 cells/μl in 2007 and 167 cells/μl in 2011; the decrease after 2011 was 4% (95% CI: 2.9%, 5.1%) annually, with a geometric annual CD4e mean of 148 cells/μl in 2014.

**Fig 2:**
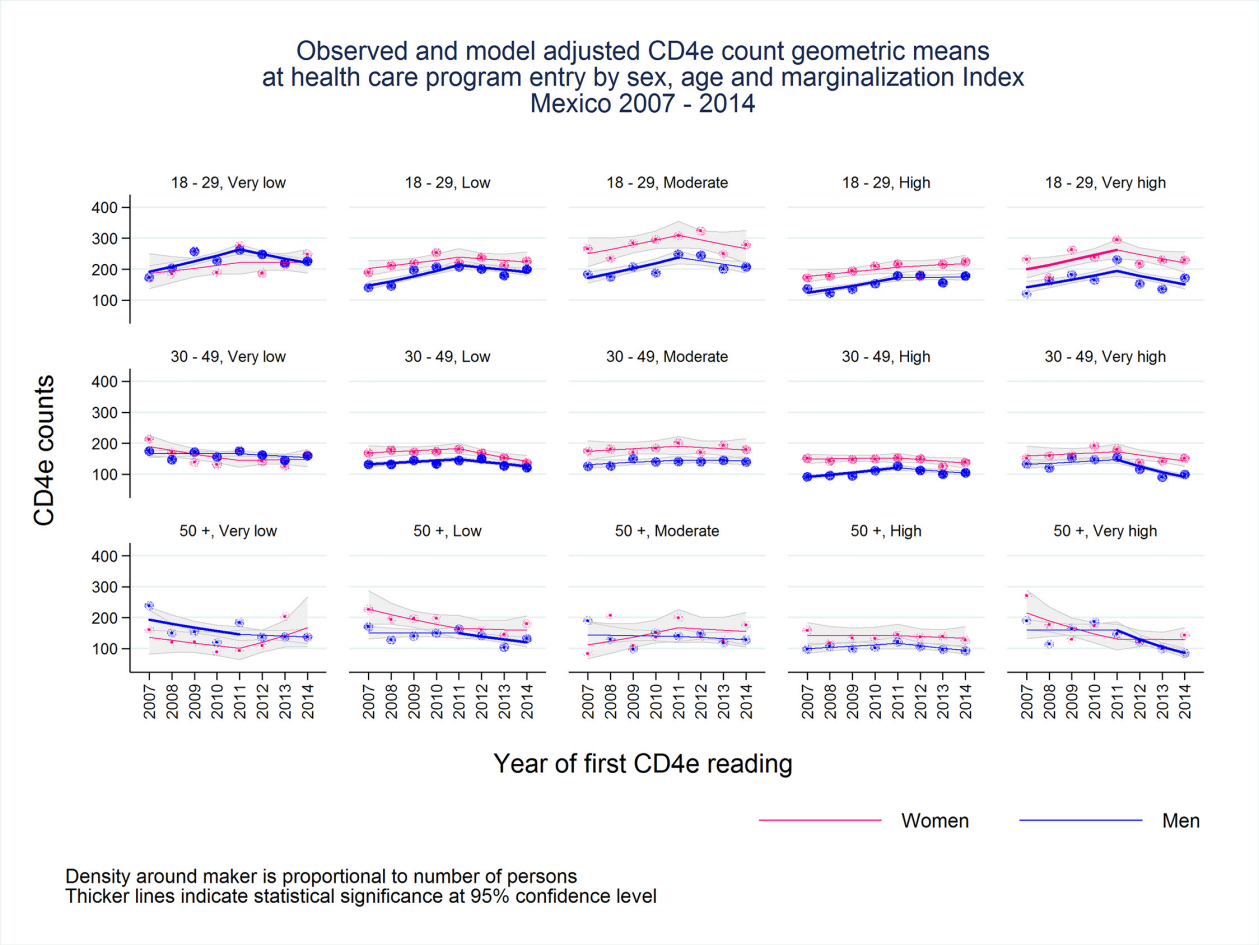
There is high variability in the tendency of CD4e counts at health care program entry between the age, sex and marginalization index groups but a decline after 2011 is observed in the majority of the groups.

Fig 2 and Table 3 summarize the results from the log linear model that considered the interaction terms for sex, age category, and MI of the state where treatment was provided in the prediction of annual geometric mean of CD4e cell counts. Women entering into HIV care had higher CD4e counts than men did; this difference appeared to be independent of the MI of the state where treatment was provided. However, this gender difference became non-significant for the age category of 50 or more years, probably reflecting the small sample size in these groups. Women and men in the younger age category entering HIV care (18–29 year old) had higher CD4e cell counts at entry.

**Table 3:** Adjusted estimates of annual percent change in geometric mean CD4e at health care program entry by sex, age category, and state MI, SALVAR, Seguro Popular, Mexico 2007–2014.

			CD4e 2007–2011		CD4e 2012–2014	
			Annual % change in geometric mean CD4e		Annual % change in geometric mean CD4e	
Sex	Age category	State MI†	Estimate	95% CI	Estimate	95% CI
Female	18–29	Very low	4.55	(-4.2, 14.1)	0.04	(-8.88, 9.84)
Female	18–29	Low	4.25	(-0.8, 9.5)	-2.26	(-8.59, 4.52)
Female	18–29	Moderate	5.43	(-0.9, 12.2)	-4.80	(-12.04, 3.05)
Female	18–29	High	4.01	(-0.1, 8.3)	1.74	(-3.80, 7.61)
Female	18–29	Very high	7.07*	**(1.8, 12.6)**	-5.67	(-11.41, 0.44)
Male	18–29	Very low	8.29*	**(4.9, 11.8)**	-5.84*	**(-8.49, -3.12)**
Male	18–29	Low	9.84*	**(6.8, 13.0)**	-3.69*	**(-6.84, -0.42)**
Male	18–29	Moderate	8.42*	**(3.8, 13.2)**	-4.75	(-9.39, 0.13)
Male	18–29	High	8.57*	**(5.5, 11.7)**	0.46	(-2.67, 3.70)
Male	18–29	Very high	8.12*	**(3.0, 13.6)**	-7.90*	**(-12.02, -3.59)**
Female	29–49	Very low	-6.43	(-12.5, 0.03)	1.07	(-8.82, 12.03)
Female	29–49	Low	2.21	(-8.75, 14.48)	-5.95	(-17.08, 6.68)
Female	29–49	Moderate	1.44	(-10.64, 15.15)	2.84	(-10.17, 17.74)
Female	29–49	High	1.05	(-9.67, 13.04)	-5.27	(-16.64, 7.66)
Female	29–49	Very high	-0.43	(-11.27, 11.74)	-0.30	(-13.05, 14.31)
Male	29–49	Very low	0.08	(-2.47, 2.70)	-2.41	(-5.38, 0.65)
Male	29–49	Low	3.15*	**(0.74, 5.62)**	-5.53*	**(-8.16, -2.83)**
Male	29–49	Moderate	2.71	(-0.89, 6.45)	-0.70	(-4.88, 3.68)
Male	29–49	High	7.44*	**(4.84, 10.10)**	-0.75	(-13.70, 14.15)
Male	29–49	Very high	2.67	(-1.21, 6.69)	-14.65*	**(-18.54, -10.57)**
Female	50 or more	Very low	-7.15	(-22.89, 11.82)	18.15	(-7.96, 51.67)
Female	50 or more	Low	-7.51	(-18.81, 5.36)	1.36	(-14.78, 20.54)
Female	50 or more	Moderate	9.59	(-10.66, 34.43)	2.53	(-16.53, 25.94)
Female	50 or more	High	0.28	(-12.39, 14.78)	-3.57	(-18.42, 13.99)
Female	50 or more	Very high	-13.87	(-26.94, 1.55)	5.59	(-13.86, 29.43)
Male	50 or more	Very low	-6.72*	**(-12.82, -0.20)**	-2.09	(-9.89, 6.39)
Male	50 or more	Low	-0.01	(-4.47, 4.67)	-7.14*	**(-13.03, -0.84)**
Male	50 or more	Moderate	-0.94	(-8.59, 7.34)	-2.46	(-11.73, 7.78)
Male	50 or more	High	4.12	(-1.72, 10.29)	-6.82	(-14.29, 1.31)
Male	50 or more	Very high	-0.14	(-7.53, 7.85)	-18.67*	**(-26.59, -9.91)**

SALVAR: antiretroviral drug administration, logistics and surveillance system; CD4e = CD4 counts at entry into care; MI = marginalization index; CI = Confidence Intervals

*P-value <0.05

†MI of the state of residence was classified according to the CONAPO marginalization index 2010, available at: http://www.conapo.gob.mx/en/CONAPO/Indices_de_Marginacion_2010_por_entidad_federativa_y_municipio

When stratifying by sex, age, and MI, we found that the annual geometric mean CD4e cell counts for women were stable over the study period with no meaningful improvements; there was, however, one exception: a small increasing trend in CD4e cell counts was observed among women aged 18–29 entering HIV care in states with very high MI during the period from 2007 to 2011 (Fig 2). In contrast, for men, the annual geometric mean CD4e cell counts showed significant increasing trends for the period 2007 to 2011 among the 18–29 and 30–49 age categories and some decreasing trends after 2011 for the same age groups (Fig 2).

## Discussion

Universal coverage of HIV care and timely ART initiation are essential to decrease HIV-related morbidity and mortality in PLHIV and to decrease community viral load, thus reducing the rate of HIV infection in the general population. We presented data on 66,947 PLHIV receiving care through public healthcare institutions financed by SP from 2007 to 2014. Through SP, the population previously excluded from HIV care and treatment coverage–the unemployed and those working in the informal sector [21]–gained access to ART and clinical monitoring at no cost. From 2010 to 2012, the Mexican government spent nearly US $400 million for ART and ambulatory care for PLHIV covered by SP.[30, 31] The increasing numbers of PLHIV covered by SP and entering care each year may reflect important advances in universal coverage for ART in Mexico, highlighting the improvements in access to HIV care and treatment in the country. However, our findings reveal that late entry into care is highly prevalent among PLHIV, with critical inequities between sex, age, and state MI.

From 2007 to 2014, the overall percentage of individuals with a late HIV diagnosis (CD4<200 cells/mm3) has declined slightly, from 52.4% to 47.6%, yet it remained above 50% in high and very high marginalization states. These figures may be underestimates, as we only considered the criterion of CD4≤ 200 and not the clinical AIDS-defining conditions, for which we lack data. Women had higher CD4e cell counts than men did, and these differences persisted after controlling for age and the MI of the state where they received care. Consequently, the proportion of those with a late diagnosis was lower among women entering into HIV care. Older adults (aged 50 and over) had a greater likelihood of being diagnosed late when compared with younger adults (56.5% vs. 38.9%, respectively).

Relative to other countries in Latin America and the Caribbean, Mexican SP-beneficiaries living with HIV/AIDS have one of the highest proportions of late entry into care. According to a 2013 report by the Pan American Health Organization, the proportion of individuals with CD4≤ 200 cells/μl ranges from 10% in Cuba to 58% in Guatemala, with a median of 40% for the region.[32] In North America, Europe, and Australia, proportions of late entry into care (CD4 <200 cells/μl or AIDS-defining conditions) range between 8.8% and 28.7% in 2009–2010 [18, 33].

We observed no net increase in the geometric mean CD4e during the study period. While a modest increase in CD4e was observed from 2007 to2011, this trend was reversed in subsequent years. The lack of increase in CD4e is not unique to Mexico: recent meta-analyses demonstrated minimal increase in CD4e during similar periods in high-income countries in North America and Europe, as well as low- and middle-income Sub-Saharan Africa.[16, 34] Other reports, however, suggest significant declines in late diagnosis, especially among younger males.[35]

The high proportion of late diagnosis among PLHIV covered by SP documented in our study may be a contributing factor to the prevalence of high national and state-level AIDS-mortality trends observed in Mexico, where AIDS mortality trends have remained stable following 2007 when ART became universally and freely available to the uninsured and SP beneficiaries.[36]

We observed a significant increase in CD4e counts ranging from 8% to 10% annually from 2007–2011 among men aged 18–29 regardless of the MI of the state where they received treatment. However, this positive trend reversed after 2011: the magnitude was lower than the net overall increase. In contrast, among men aged 30 and above, our results suggest that CD4 cell counts at entry to care have remained stable, and there are several possible explanations for these findings. First, the expansion of SP may have helped previously uninsured young men access care and treatment. Second, young adults are more involved in social networks and health promotion circles, which may influence their care-seeking behaviors. Third, it is possible that HIV testing among younger individuals has significantly increased; there is no evidence, however, to support this claim. A recent study in Mexico City found that the proportion of individuals entering care who were diagnosed with HIV before the age of 25 significantly decreased from 1995–2012, highlighting that even if younger individuals are diagnosed early they may be delaying their entry into care.[37] Results from our study are consistent with this observation, with older individuals entering care with very low CD4 cell counts. Although we were unable to assess whether late entry into care was due to delayed testing or delayed presentation to care, our findings highlight the importance of promoting testing among older individuals who may be considered lower risk by providers, and who may not perceive the extent of their risk for HIV.

Sex disparities were prominent in our study. With the exception of women residing in very low MI states, CD4e levels were generally higher among women than men at entry into care; yet we only observed an increasing trend in CD4e from 2007–2011 among women aged 18–29 living in very high MI states. While the prevalence of HIV among women is low in the general population, the distribution of PLHIV in our study may indicate a disproportionate burden of HIV among women in high and very high MI states, where male-to-female ratios were lower. Our observations are consistent with findings of heterosexual transmission from migrants returning from the US to their rural communities [38] and highlight the need to improve awareness and prevention of HIV in these regions. In addition, the increase in CD4e observed among women aged 18–29 in high MI states may be explained by high fertility rates combined with the presence of conditional cash-transfer programs in these states: they provide improved prenatal care and access to HIV testing programs. Although great strides have been made to increase prenatal HIV testing, suboptimal HIV testing practices persist. A recent study in central Mexico highlighted that despite multiple visits to health providers, less than half of women were offered HIV tests, even when presenting with AIDS-related complications.[39] There is a crucial need to improve the ability of healthcare providers to identify HIV risk and implement testing protocols, especially during pregnancy. Furthermore, the lower CD4e levels among women in very low MI states relative to men highlight the existence of sex disparities, even in affluent regions of the country. Women living with HIV in these states frequently have social and economic vulnerabilities that put them at higher risk and require specific HIV prevention and testing strategies.[40]

Previous research showed that late testing is a major contributor to late entry into care in Mexico.[41] This finding is complemented by evidence of low proportions of HIV-testing in the general population, with 21% of men and 23% of women aged 20–49 years reporting ever being tested for HIV in 2012[42]; among MSM, 46% report having been tested for HIV in the previous 12 months[43]. Yet data are scarce regarding correlates of late HIV testing: previous studies in Mexico have identified unemployment, older age, and low educational level as demographic predictors of late testing, while reasons for not testing for HIV include preferring not to know that one has HIV and having peers who engage in high-risk behavior.[20] In a representative venue-based sample of 4,224 MSM not tested for HIV in the previous 12 months, 37% said they did not feel at risk of HIV, 19% reported fear of receiving HIV-test results, and 12% said they did not knowing where to get an HIV test.[43] In addition, stigma and discrimination may also play roles in late testing. A study of health care providers in Mexico found that 78% of physicians considered themselves at risk for HIV-infection when treating a patient with HIV, and 75% blamed the patient for becoming infected with HIV.[44] In 2012, among a nationally representative survey of MSM, 56% of those surveyed said they had felt stigmatized and discriminated against during the previous year, with higher proportions in states of high and very high marginalization.[43] More research is needed to identify the structural, social, and psychological barriers to HIV testing, and to develop strategies to combat stigma, as well as effective methods of communicating with higher-risk groups who may not consider themselves at risk for HIV.

Mexican HIV testing guidelines constitute an important structural barrier to early diagnosis. Currently, rapid HIV testing procedures require that a separate consent form be signed prior to any HIV test and that counseling takes place before and after testing.[45] Evidence suggests that removing requirements for separate written consent and providing an opt-out strategy to HIV testing actually increases HIV testing rates among the general population in health care settings.[46–48] In addition, although pre- and post-testing counseling has been an integral part of rapid HIV testing, the benefits of coupling HIV prevention counseling to rapid HIV testing practices have recently been contested.[49] Modifying the current rapid HIV testing policy to make it less resource-intensive may facilitate early detection of HIV and allow the introduction and evaluation of more innovative strategies to increase testing, such as offering home-based rapid HIV testing.[50]

The main strength of our study is its basis in a comprehensive national surveillance database with high data quality and completeness. In addition, the database is representative of approximately 60% of the population receiving HIV care in Mexico. Nevertheless, our study is subject to several limitations. First, although we attempted to minimize information bias by including only ART-naïve individuals, it is possible that those individuals who had initiated ART before enrolling in SP were included in our analysis. However, this would have biased our estimates towards higher CD4e levels, suggesting that the low CD4e levels observed may be even lower. Second, given that CD4 tests were out-of-pocket expenses for SP enrollees prior to 2009, it is possible that we included only a subset of those new patients with greater financial resources entering care in 2007 and 2008; this inclusion may serve as a source of bias in our estimation of time trends. Given that CD4 tests prior to 2009 were paid out-of-pocket by patients, however, the direction of the bias is likely towards the higher CD4 cell counts, as people able to afford CD4 tests may have had increased access to HIV care and may have sought care at an early stage of the disease. Third, CD4 tests were not processed at a single laboratory and there is no standard protocol in Mexico for accreditation of a laboratory’s competence; thus, both random and systematic error may be present in our estimates. Fourth, the state marginalization index is an ecological measurement; therefore, while it may relate to characteristics of the health care system and patterns of demand and delivery of services, it does not inform on individual socioeconomic status.

## Conclusions

Our data suggest that the HIV epidemic in Mexico continues to grow, as indicated by the number of new patients entering into SP-HIV care programs each year. The creation of SP has resulted in a significant expansion of HIV care and treatment, with increasing numbers of PLHIV entering care and accessing ARV treatment, at no cost at the point of care. However, late entry to HIV care remains an important barrier to improving the effectiveness of the universal- access ARV program. In spite of significant efforts, the frequency of late entry into ARV treatment remained relatively unchanged through the 2007–2014 period. In 2014, 47.6% of new patients entering into HIV-care had CD4 cell counts below 200 cells/μl. The geographic, socioeconomic, and demographic differences in CD4e cell counts highlight areas for improvement in Mexico’s HIV prevention, care, and treatment services.

Our results also highlight the urgent need to reverse late entry into care as a critical strategy to improve the cost-benefit ratio of SP´s universal ARV access program. Reducing the time between HIV infection and presentation for treatment is key to reducing the heightened economic burden of the ARV program and to ensure the sustainability of the program.

In addition, our results underline the importance of accessing information systems with timely CD4 surveillance data to monitor late diagnosis as a critical public health outcome for assessing AIDS prevention and treatment programs in Mexico.
